# Danger in Large Doses of Quinine

**Published:** 1888-12

**Authors:** 


DANGER IN LARGE DOSES OF QUININE.—Quinine sometimes affects the eyes dangerously. He relates the case of a man who came to him from Ohio, several years ago, totally blind.

This man had, in the attempt to doctor himself for an attack of malaria, taken two doses of quinine, an hour apart, each consisting of a heaping tablespoonful of the drug. Blindness followed in a few hours; and treatment proved of no avail; the man's sight is still wanting.—*Keyser.*

